# MCSS: microbial community simulator based on structure

**DOI:** 10.3389/fmicb.2024.1358257

**Published:** 2024-03-07

**Authors:** Xingqi Hui, Jinbao Yang, Jinhuan Sun, Fang Liu, Weihua Pan

**Affiliations:** ^1^Zhengzhou Research Base, State Key Laboratory of Cotton Biology, School of Agricultural Sciences, Zhengzhou University, Zhengzhou, China; ^2^Shenzhen Branch, Guangdong Laboratory of Lingnan Modern Agriculture, Genome Analysis Laboratory of the Ministry of Agriculture and Rural Affairs, Agricultural Genomics Institute at Shenzhen, Chinese Academy of Agricultural Sciences (ICR, CAAS), Shenzhen, China; ^3^College of Informatics, Huazhong Agricultural University, Wuhan, China; ^4^Key Laboratory of Plant Molecular Physiology, CAS Center for Excellence in Molecular Plant Sciences, Institute of Botany, Chinese Academy of Sciences, Beijing, China; ^5^National Key Laboratory of Cotton Bio-Breeding and Integrated Utilization, Institute of Cotton Research, Chinese Academy of Agricultural Sciences (ICR, CAAS), Anyang, China

**Keywords:** metagenome, microbiome communities, long reads, simulator, assembly

## Abstract

*De novo* assembly plays a pivotal role in metagenomic analysis, and the incorporation of third-generation sequencing technology can significantly improve the integrity and accuracy of assembly results. Recently, with advancements in sequencing technology (Hi-Fi, ultra-long), several long-read-based bioinformatic tools have been developed. However, the validation of the performance and reliability of these tools is a crucial concern. To address this gap, we present MCSS (microbial community simulator based on structure), which has the capability to generate simulated microbial community and sequencing datasets based on the structure attributes of real microbiome communities. The evaluation results indicate that it can generate simulated communities that exhibit both diversity and similarity to actual community structures. Additionally, MCSS generates synthetic PacBio Hi-Fi and Oxford Nanopore Technologies (ONT) long reads for the species within the simulated community. This innovative tool provides a valuable resource for benchmarking and refining metagenomic analysis methods.

**Code available at:** https://github.com/panlab-bio/mcss

## 1 Introduction

Metagenomic sequencing treats microbes in the environment as a unified entity to obtain genomic sequences, which can be used to study the taxonomic composition of microbial communities and identify novel species (Handelsman et al., 1998; Handelsman, 2004; Chen and Pachter, 2005; Frioux et al., 2020; Yang et al., 2021). And the assembly of metagenomic sequencing reads into metagenome-assembled genomes (MAGs) is a crucial step in the metagenomic analysis. Assembly tools, such as hifiasm_meta (Feng et al., 2022) and metaFlye (Kolmogorov et al., 2020) enhance contiguity in assemblies using nanopore (Wang et al., 2021) and PacBio (Rhoads and Au, 2015) sequencing data compared to short-read assemblies. And they can effectively address challenges of uneven species composition and intra-species heterogeneity in complex microbial communities (Marx, 2021; Bickhart et al., 2022). The development and testing of these metagenome assembly algorithms require high-quality benchmark datasets with ground truth, but obtaining ground truth for real datasets can be challenging, making it difficult to assess the accuracy of the algorithms (Escalona et al., 2016; Zhao et al., 2017; Alosaimi et al., 2020). Therefore, the development of simulation software that can generate synthetic metagenomic data is highly meaningful.

So far, several simulation tools have been developed. Read simulators like Pbsim3 (Ono et al., 2022) and NanoSim (Yang et al., 2017) can generate simulated third-generation sequencing reads, which provide foundational data for benchmark testing. However, they cannot simulate metagenomic data. Meta-NanoSim (Yang et al., 2023) and CAMISIM (Fritz et al., 2019) can simulate metagenomic datasets but require users to provide additional information, such as a reference metagenome list or the composition of the microbial community. This requires users to have a prerequisite level of domain expertise, so in many cases, users may not be certain about the species composition of the microbial community they want to simulate. M&Ms García-García et al. (2022) can simulate datasets based on environmental parameters, allowing users to specify the environment they want to simulate and obtain simulated metagenomes. Since M&Ms acquire species within genera through random sampling, it does not consider the structural characteristics of communities at the species level and cannot learn from the characteristics of the real sequencing samples entered by the user. Additionally, the sequencing data simulated by M&Ms. is limited to 16S rDNA and cannot generate third-generation sequencing data for whole genomes.

Therefore, we have developed MCSS, which can simulate microbial communities and generate third-generation sequencing data. MCSS generates simulated data based on community structure at the species level, preserving the structural features of real samples while expanding the diversity of the simulated community. Moreover, MCSS can simulate both the abundance of species within the community and intra-species heterogeneity, which increases the complexity of the simulated data, making it more closely resemble real samples. Finally, the generated long reads can be used directly as input for assembly tools, greatly reducing the workload for users.

## 2 Materials and methods

**Figure 1:**
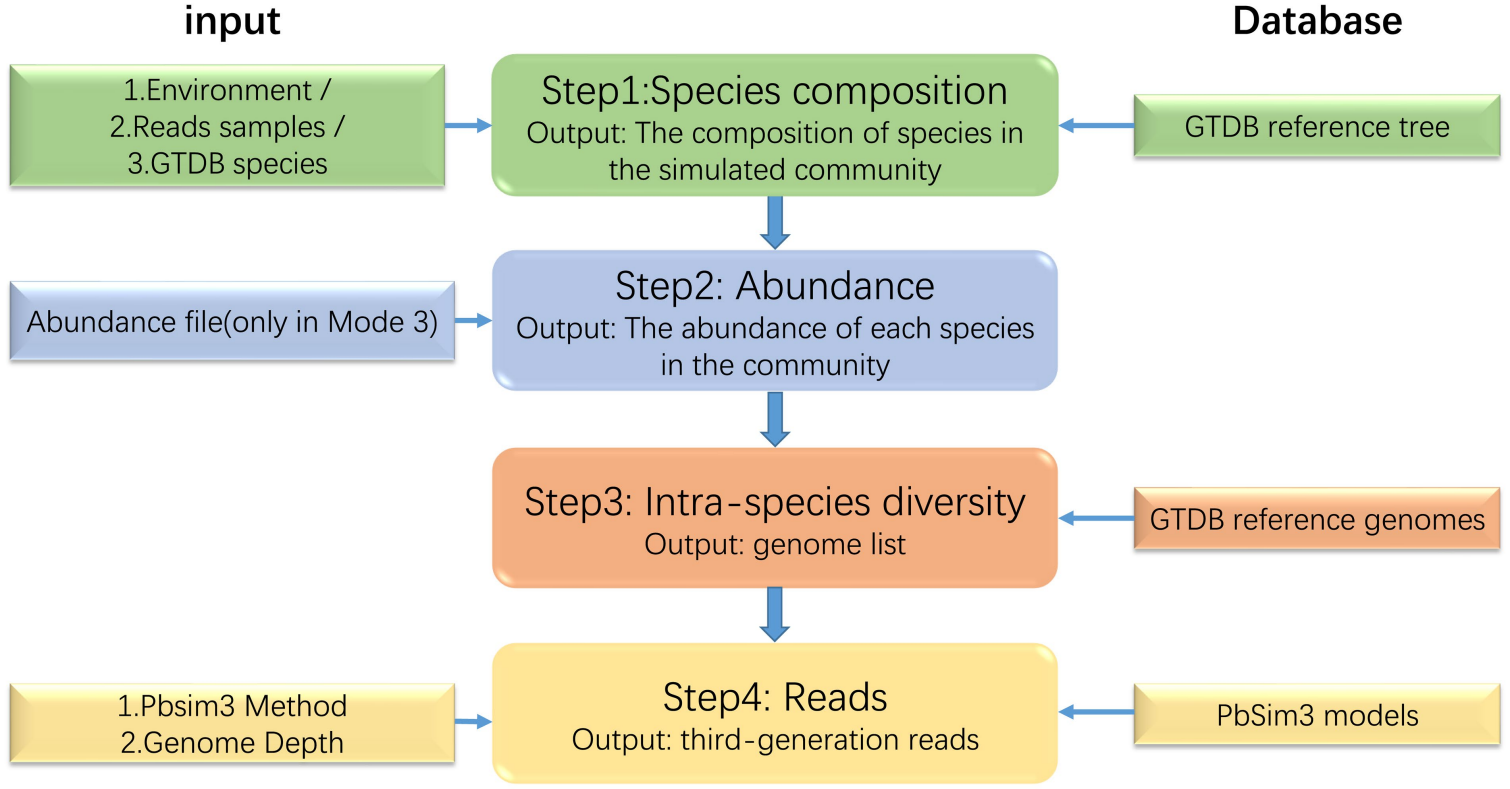
Flow diagram of the MCSS workflow.

MCSS generates simulated microbial communities and sequencing data by learning the structure, abundance, and intra-species heterogeneity information from real samples of microbial communities. MCSS primarily generates simulated data through the following four steps (Figure 1): (1) determine the species composition, (2) determine the abundance of each species in the community, (3) find the reference genomes of the species in the GTDB reference database (Parks et al., 2020), and (4) call Pbsim3 to generate simulated long reads.

**Figure 2:**
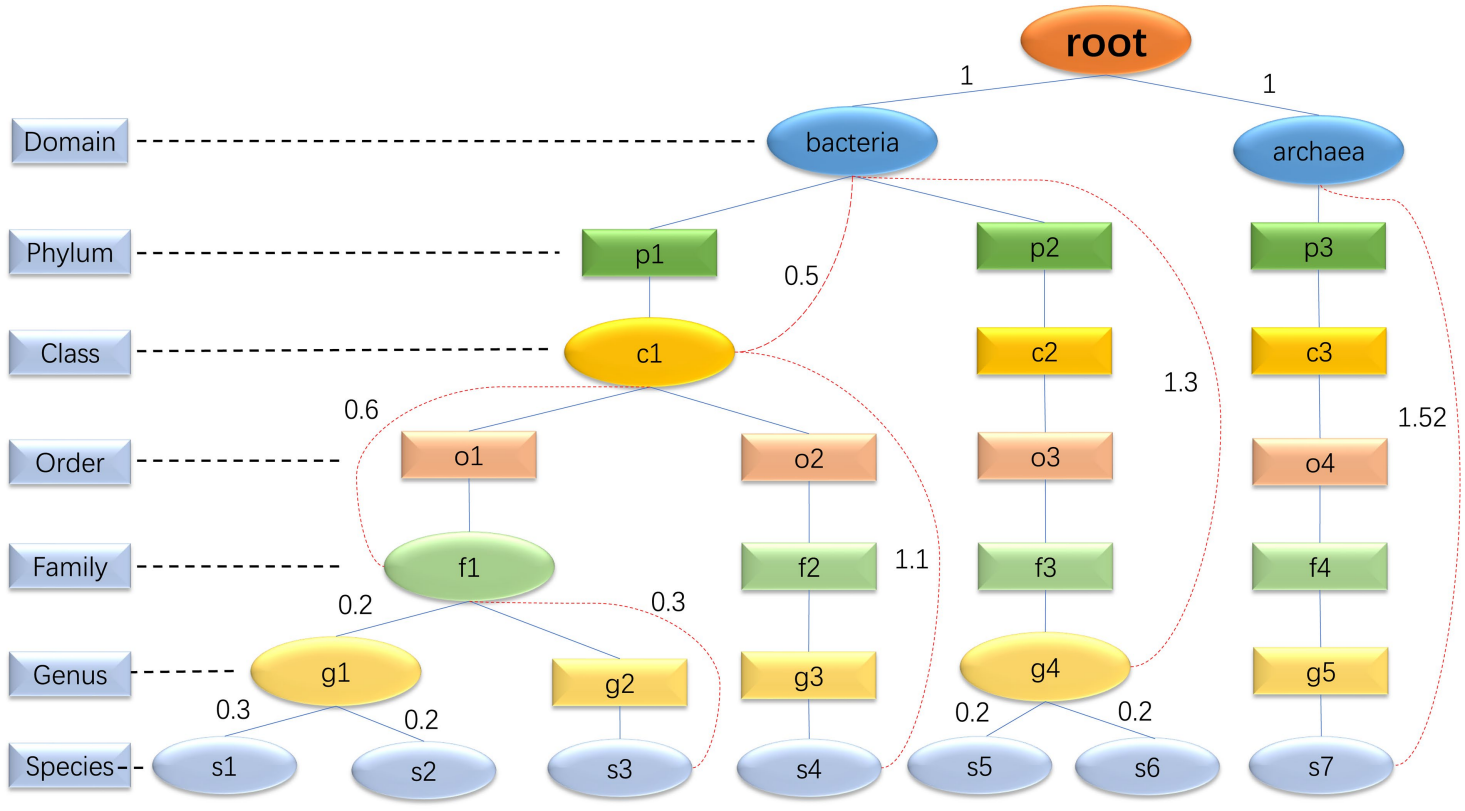
Multiway trees: the diagram is an illustration of a microbial community multiway tree, where oval nodes represent entity nodes, and rectangular nodes represent non-entity nodes. The evolutionary distance between entity nodes can be calculated, but it is not possible to calculate the evolutionary distance between entity nodes and non-entity nodes, or between non-entity nodes themselves. For example, the genus g2 only has one species, s3. Although g2 can be mapped to a specific taxonomic unit in the GTDB database, the information in the GTDB database about this genus corresponds to s3 rather than the genus itself. Consequently, it is not possible to calculate the evolutionary distance from other nodes to g2. Hence, we consider g2 as a non-entity node. All domains and species are entity nodes. For other taxonomic hierarchy levels, a node is considered an entity node if it has two or more directly adjacent child nodes; otherwise, it is considered a non-entity node. The red dashed lines are used to connect a parent entity node and its non-directly adjacent child node to record the evolutionary distance between these two nodes. The evolutionary distance between two directly adjacent entity nodes is directly represented by the edge length.

The core function of step 1 is to determine the species composition of the simulated microbial community based on real samples. In the community, each species can be mapped to a corresponding taxonomic rank (domain, phylum, class, order, family, genus, and species) within the GTDB database. This taxonomic rank resembles a branch, and all the species’ taxonomic ranks form a tree. High-level taxonomic units may have one or more subordinate low-level taxonomic units. Consequently, we have decided to represent the taxonomic profiles of microbial communities using a multiway tree structure, which is a data structure allowing multiple branches for each node (Figure 2). In our study, we use a multiway tree to represent the structural characteristics of a microbial community and construct a multiway tree for each real sample’s community. Then, we construct a multiway tree based on all species in the GTDB reference database using the same method, to serve as a reference multiway tree. For each simulation, we sample a multiway tree from real samples, and then identify the optimal subtree of the sampled multiway tree within the reference multiway tree, representing the species composition of the simulated community. We have pre-generated community multiway trees for multiple samples under various environmental conditions (Table 1), which serve as the basis for sampling and creating the sampled multiway trees. Furthermore, MCSS can produce sampled multiway trees using user-input sequencing data.

**Table 1:** The sources and quantities of actual samples from different environments.

Environment	Project	Sample count
Gut	PRJNA398089	104
Soil	PRJNA252425	118
Oral	PRJNA362687	111
Skin	PRJEB26427	102
Marine	PRJNA329908	123
Rhizosphere	PRJEB23682	120

In the second step, the abundance of each species in the community is determined by sampling based on species abundance observed in real samples. Because not all community species abundance distributions can be fitted with appropriate models, sampling from real samples is widely applicable across various environments.

In the third step, MCSS searches the GTDB reference database for the genomes of each species in the community. If the user specifies the number of strains within each species, the tool will search for that specified quantity of genomes for each species to reflect the diversity between species.

In the final step, the user needs to specify the minimum depth of coverage or average depth of coverage, as well as the sequencing model for Pbsim3. MCSS then calls Pbsim3 to generate simulated sequencing data. By using simulated community genome list as input, Pbsim3 simulation allows the generation of synthetic metagenomic sequencing data. Pbsim3 can generate both high-accuracy Hi-Fi reads and ultra-long ONT reads, with sequencing costs higher than those associated with second-generation sequencing reads. In addition, we provide the function to exclusively generate simulated community genome data. Users can choose a sequence simulation tool that suits their research to generate sequencing reads for simulated genomes.

### 2.1 Determine the species composition

#### 2.1.1 Data sources

We downloaded sequencing data for six environmental conditions (Table 1) from the MGnify (Richardson et al., 2023) and NCBI SRA (Sayers et al., 2023) database, which include the gut (PRJNA398089) (Zhang et al., 2022), marine (PRJNA329908) (Tremblay et al., 2017), oral (PRJNA362687) (Qiao et al., 2018), rhizosphere (PRJEB23682) (Maarastawi et al., 2018), skin (PRJEB26427) (Lam et al., 2018), and soil (PRJNA252425) (Větrovský et al., 2020). For each environment, we downloaded multiple samples where, each set of sequencing data represents a single sample.

#### 2.1.2 Real and reference multiway tree

We assign GTDB taxonomic labels (Youngblut and Ley, 2021) to sequencing reads with Kraken2 (Wood et al., 2019) (GTDB_release207) and retrieve the taxonomic information (kingdom, phylum, class, order, family, genus, and species) of each species in the samples from the GTDB reference database for constructing the multiway tree of the microbial community structure (Figure 2). Each node in the multiway tree (T_real) represents a taxonomic unit. The edge lengths are determined by calculating the evolutionary distance in the GTDB reference tree, representing the evolutionary distance between two taxonomic units. Construct a multiway tree based on all species in the GTDB reference database using the same method, to serve as a reference multiway tree.

#### 2.1.3 Simulated multiway tree

We randomly sample the multiway trees of environmental samples to obtain the sampled multiway tree. In the reference multiway tree, use Algorithm 1 to identify the subtree that closely resembles the sampled multiway tree. The essence of Algorithm 1 lies in finding a subtree in the reference multiway tree such that the difference in evolutionary distance between this subtree and the sampled multiway tree is minimized, which can be addressed using recursion. The species within this subtree constitute the simulated microbial community. To make MCSS more practical, users can specify two search modes to find subtrees, which are the accurate mode and the prolific mode. In the accurate mode, the search in reference multiway tree is based solely on sampled multiway tree, while in the prolific mode, adjustments are made using the mean and standard deviation of evolutionary distances for species in the sample to expand the simulated data. The process of calculating evolutionary distance is described by the following formula (formulas (1), (2), (3), (4), and (5)):


(1) p=B(1,0.5)

(2) σ=std(dreal)

(3) u=mean(dreal)

(4) dnref={y∈dref|u−σ≤y≤u+σ}

(5) $$dsam_{g}=\{\begin{array}{c} dsam_{gs}\sim dreal_{g},s\in sam_{g};accmode\\ dsam_{gs}\sim \{\begin{array}{c} dreal_{g},s\in sam_{g},p=1\\ dnref_{g},s\in sam_{g},p=0 \end{array};prolmode \end{array}$$

where B(1,0.5) represents a Bernoulli distribution with parameters 1 and 0.5; dref , dsam , and dreal respectively represent the evolutionary distances of species within the GTDB database, sampled data, and real samples; dnref is the result of filtering dref based on dreal ; $dreal_{g}$ and $dnref_{g}$ represent the evolutionary distances of species within the genus g; $sam_{g}$ represents the set of species within genus g in the sampled data.

When the user provides FastQ files, Kraken2 is used to assign GTDB taxonomic labels to the reads in the FastQ files, and then real multiway trees are constructed.

##### ALGORITHM 1 Get_SubTree (T_sample, T_ref): Recursively search for the closest subtree.

**Input:**
 sampled multiway tree T_sample, reference multiway tree T_ref
**Output:** simulated multiway tree T_sim
If T_sample has two layers:
if prolific mode:
adjust T_sample
calculate evolutionary distance difference between species in T_sample and T_ref
return dis, node_list
Else:
min_dis = INF
choice_node = []
for child_node_T_sample in T_sample.child_nodes
for child_node_Tref in in T_ref.child_nodes
dis_tree, node_list = get_subTree(T_sample_child, T_ref_child)
if min_dis > dis_tree and child_node_Tref not chosen
min_dis = dis_tree
choice_node.append(node_list)
return min_dis, choice_node

### 2.2 Determine the abundance of each species in the community

For a specific environment, we analyze and record statistics on the abundance of species in each sample based on the Kraken2 results. Then, based on the number of species in the simulated multiway tree, we sample and normalize to obtain the abundance of microbial community species. Since the normalization process can affect the abundances of sampled species, we ensure that the sum of the sampled results approximates 100%, mitigating the impact of normalization on abundances.

When the input file consists of FastQ reads, we fit the species abundance distribution using a log-normal distribution and then sample to obtain the community species abundance.

Users can either specify both the species in a community and their respective abundances or select a pre-learned environment to generate species abundances based on that environment.

### 2.3 Find the reference genomes of the species

We downloaded multiple genomes for each species based on the correspondence between species and accession numbers in the GTDB database. These genomes are used to represent different strains within the same species. For each species in the previously determined simulated community, we randomly select the user-specified number of strain genomes. We select multiple reference genomes for a species because real samples often exhibit genetic variation within the same species. To capture these intra-species differences, we consider all distinct genomes classified under the same species when selecting species genomes. This approach ensures that the simulated genome dataset incorporates internal variations within species, facilitating an effective evaluation of the performance of metagenomic tools in handling highly similar genomes.

### 2.4 Generate simulated long reads

Since Pbsim3 (using the qshmm model by default and other error models can also be chosen) generates PacBio continuous long reads (CLR), we employ SAMtools (Danecek et al., 2021) to convert the SAM format data produced by Pbsim3 into BAM format data. Subsequently, we utilize CCS (Wenger et al., 2019) to generate PacBio high-fidelity (Hi-Fi) reads.

## 3 Results

To ascertain the extent to which the simulated tree accurately represents the structural characteristics of actual microbial communities, we randomly choose 30 samples for the test set and use the remaining ones for the training set and executed the subsequent validation procedure.

### 3.1 Consistency in the structural characteristics

**Figure 3:**
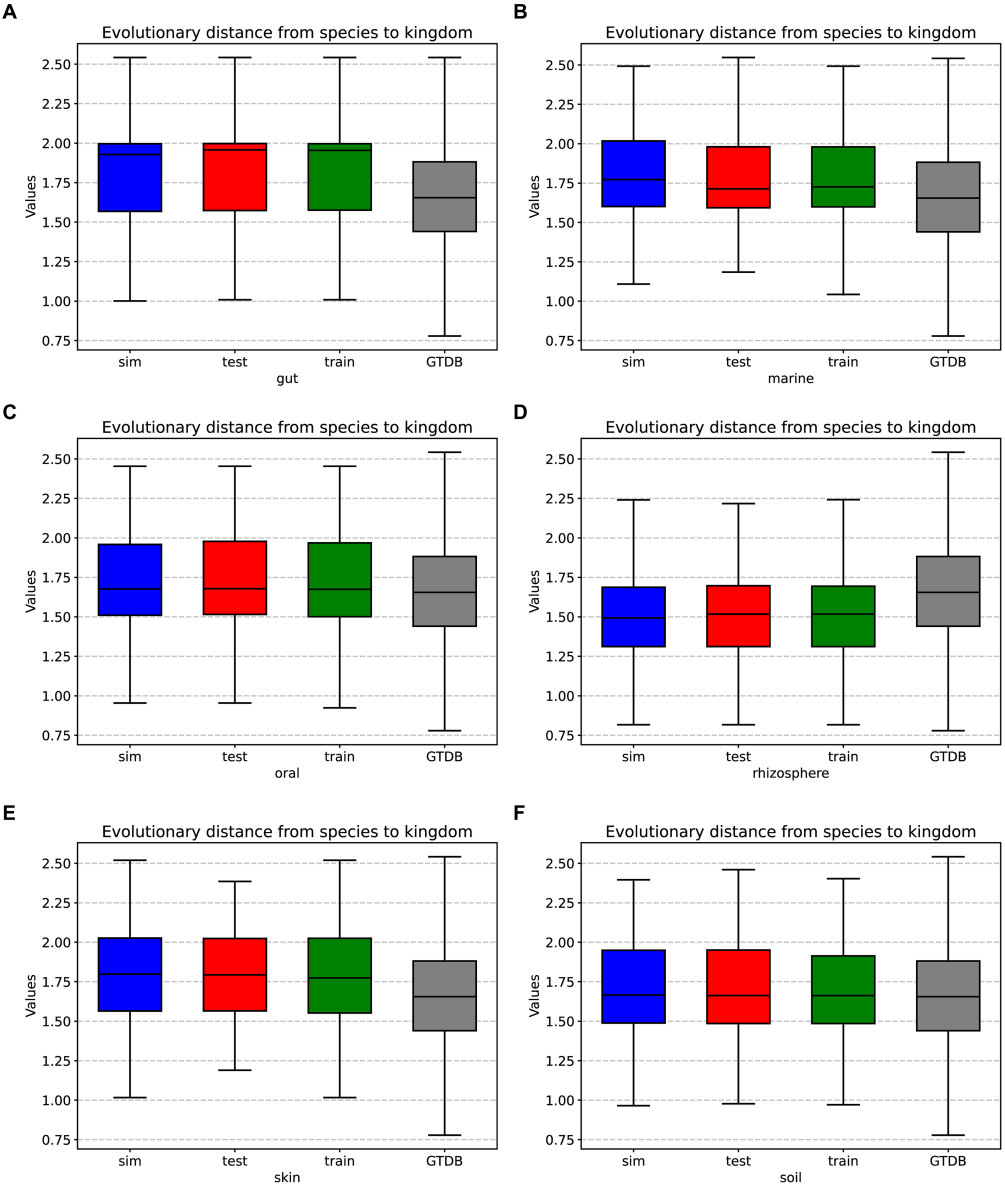
Box plots displaying the evolutionary distances from species to kingdom in the training set, test set, simulated data from different environments, and the GTDB reference database: **(A)** gut: 30 test samples and 74 training samples, **(B)** marine: 30 test samples and 93 training samples, **(C)** oral: 30 test samples and 81 training samples, **(D)** rhizosphere: 30 test samples and 90 training samples, **(E)** skin: 30 test samples and 72 training samples, **(F)** soil: 30 test samples and 88 training samples.

**Figure 4:**
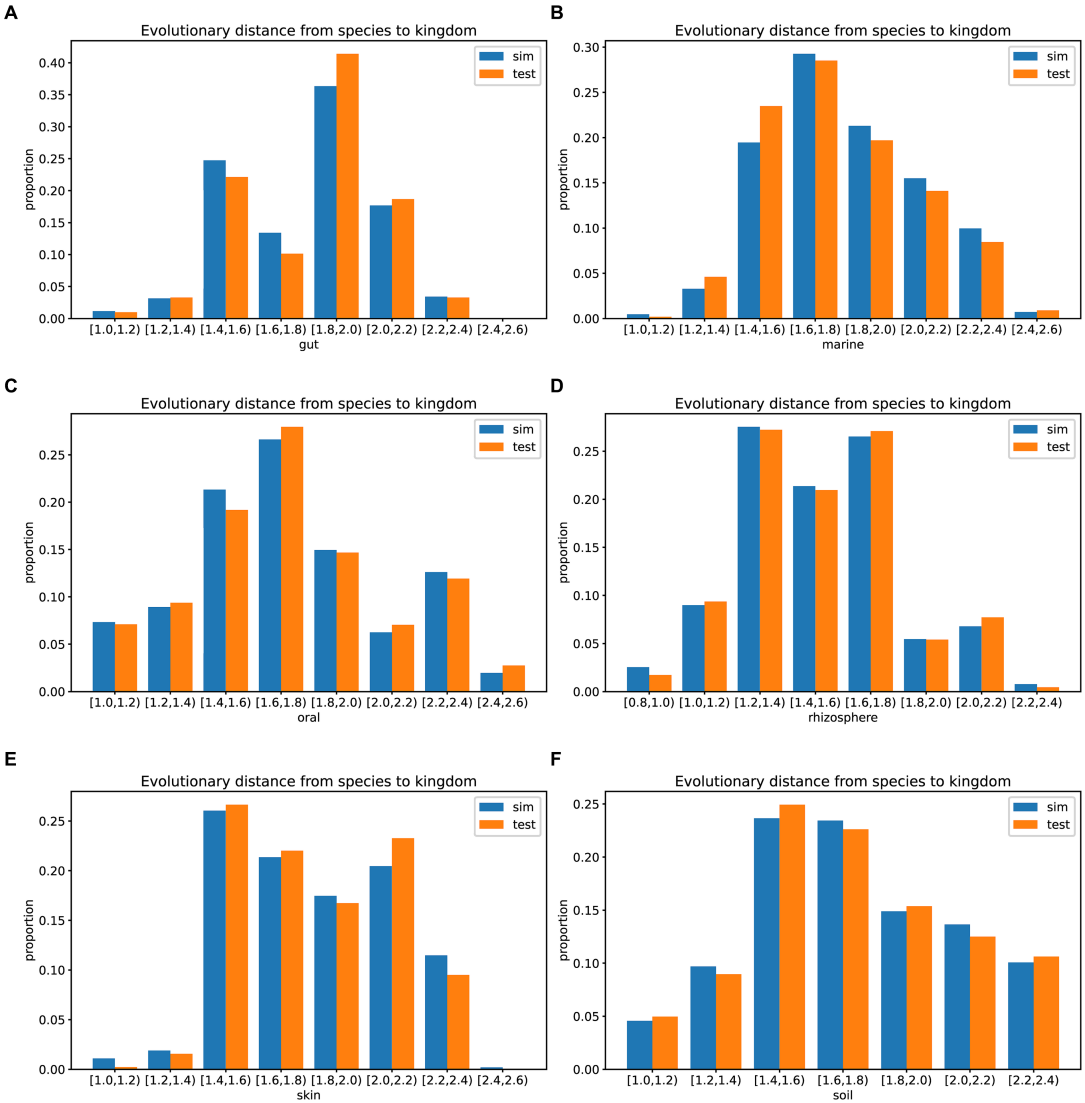
Histograms showing the evolutionary distances from species to kingdom in the training set, test set, simulated data from different environments, and the GTDB reference database: **(A)** gut: 30 test samples and 74 training samples, **(B)** marine: 30 test samples and 93 training samples, **(C)** oral: 30 test samples and 81 training samples, **(D)** rhizosphere: 30 test samples and 90 training samples, **(E)** skin: 30 test samples and 72 training samples, **(F)** soil: 30 test samples and 88 training samples.

In prolific mode, we generated 30 simulated samples using the features learned from the training dataset. We analyzed the evolutionary distances from species to kingdom in the training set, test set, simulated data, and the GTDB reference database. The species in the environment are a subset of the species in the GTDB database, reflecting the community characteristics of that environment. The community simulation process involves searching for species in the overall GTDB database that match the environmental characteristics. Figures 3, 4 show that species evolutionary distance distributions in different environments have distinct features, and they are highly consistent between the simulated and test data in each environment. The results indicate that MCSS has captured the feature of species evolutionary distances within the community from the environment.

### 3.2 Species abundance in different environments

**Figure 5:**
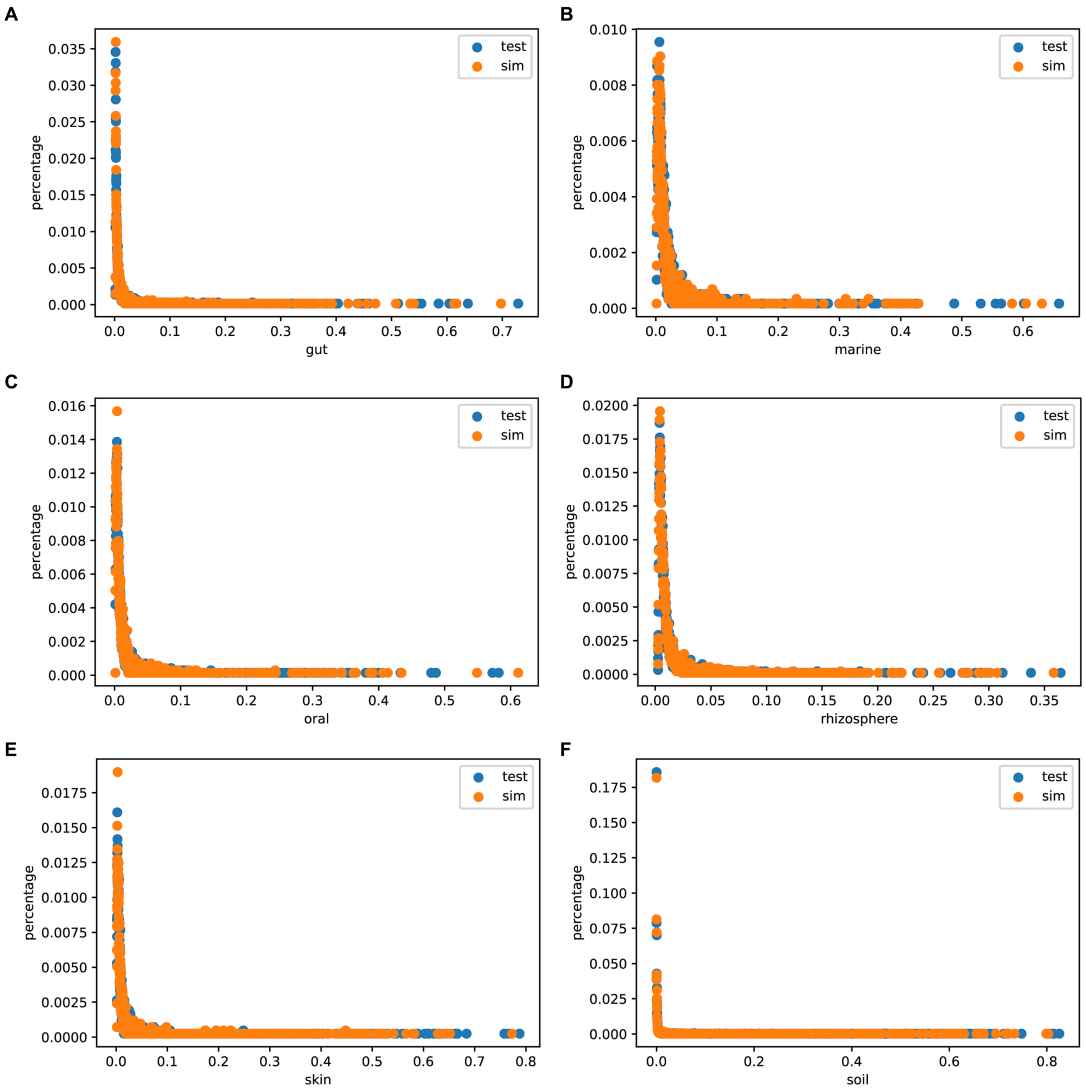
Species abundance scatter plot. The *x*-axis (0 < *x* < 1) represents species abundance, and the *y*-axis (0 < *y* < 1) represents the proportion of that abundance appearing in the samples: **(A)** gut: 104 samples, **(B)** marine: 123 samples, **(C)** oral: 111 samples, **(D)** rhizosphere: 120 samples, **(E)** skin: 102 samples, and **(F)** soil: 118 samples.

The Species abundance is a crucial metric of microbial communities. To assess the authenticity of the species abundance generated by MCSS, we compared the abundance distribution between the simulated data and the test data, and plotted scatter diagrams. Figure 5 shows that the sampled species abundances closely match the species abundances in the real samples. In environments like marine, oral, and rhizosphere, outliers are noticeable, and sampling from real samples can capture this feature, while obtaining species abundance from a distribution function fails to capture these characteristics. These results indicate that MCSS has the capability to generate relatively realistic species abundance data.

### 3.3 The consistency and diversity of the species composition

**Figure 6:**
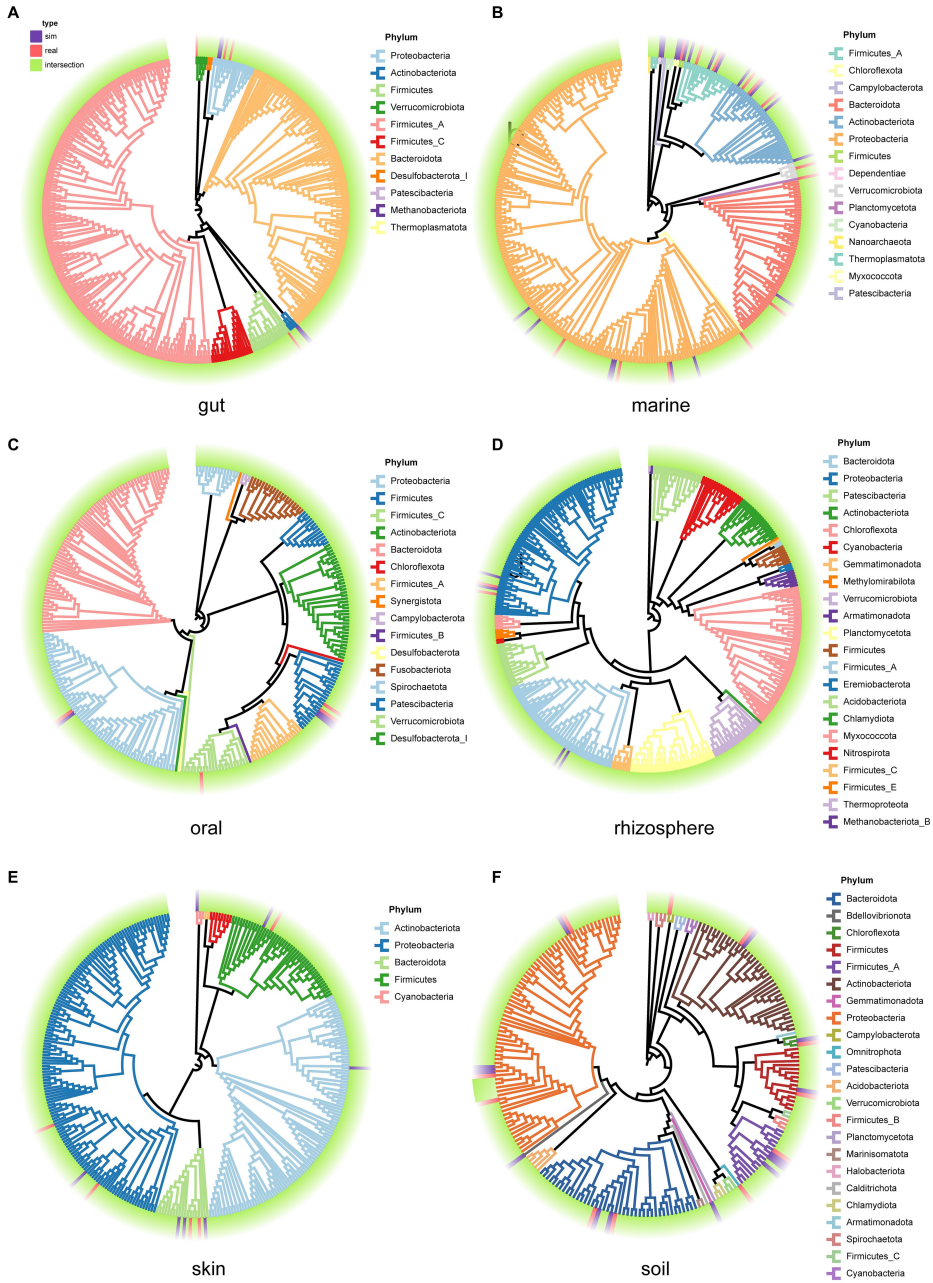
Phylogenetic trees for the species in both the real samples and the simulated samples from each environment. Purple blocks represent species that appear only in the simulated samples, red blocks represent species that appear only in the real samples, and green blocks represent species that are present in both real and simulated samples. The branch colors represent the phylum categories: **(A)** gut, **(B)** marine, **(C)** oral, **(D)** rhizosphere, **(E)** skin, and **(F)** soil.

We examined the species composition of real samples and simulated samples under user-input sample patterns. Selecting 30 samples from each environment, we generate simulated data for each sample, and then compare the species composition between the 30 real samples and the simulated samples. This process is used to evaluate the performance of MCSS in generating simulated data based on user-input samples. Compare the phylogenetic trees (Xie et al., 2023) for the species in both the real and simulated samples from each environment (Figure 6). In each environment, a high degree of overlap between the species in the simulated samples and the species in the real samples is evident. Meanwhile, there is a slight difference in the species composition between the simulated communities and the real datasets, suggesting MCSS can capture real community species composition characteristics while introducing diversity.

### 3.4 The assembly results of both real and simulated data

We generated simulated data based on SRR15275210 (Kim et al., 2022), assembled both real and simulated data separately, and analyzed the results. Despite the reduced read count in the simulated data (which can be increased by adjusting coverage), the outcomes of high-quality contigs do not differ significantly compared to real data. This is particularly evident in the number of contigs exceeding 1 M (see Table 2).

**Table 2:** The assembly results of both real and simulated data.

Result	Real	Sim
Reads	15,240,116,452	6,707,226,299
Species	160	160
Contigs with a size >500 K	244	162
Contigs with a size >1 M	128	118

### 3.5 Coverage of genomes in different environments

**Figure 7:**
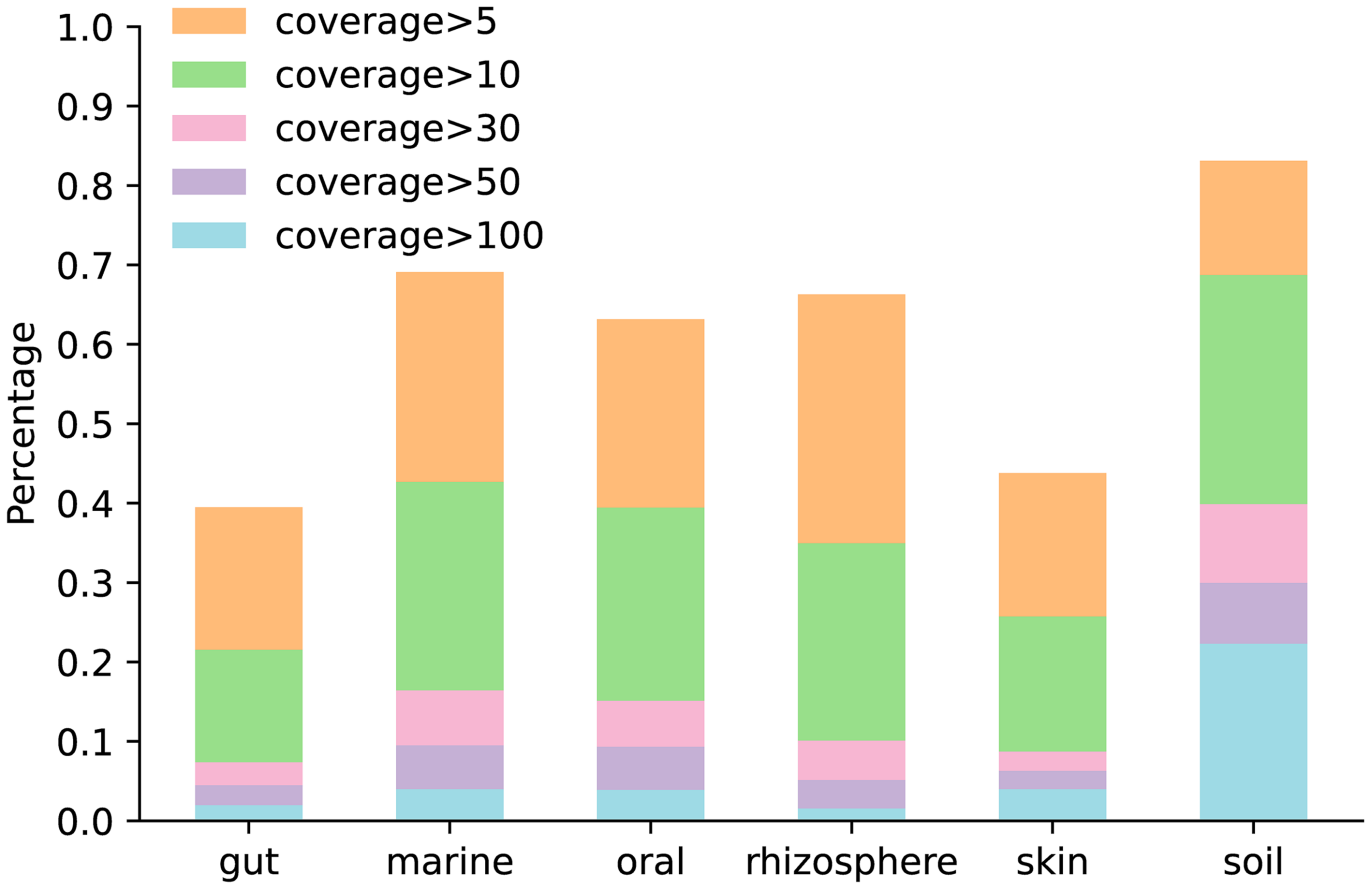
Proportion of genome coverage at various levels in different environments: the bar chart in orange and beneath represents the number of genomes with coverage greater than 5. The bar chart in green and beneath represents the number of genomes with coverage greater than 10. The bar chart in pink and beneath represents the number of genomes with coverage greater than 30. The bar chart in purple and beneath represents the number of genomes with coverage greater than 50. The bar chart in blue represents the number of genomes with coverage greater than 100.

The coverage of the genome is a critical metric, which influences the quality of the assembly. To analyze the genome coverage in simulated data generated by MCSS, we generated five simulated datasets for each environment under default parameters. Figure 7 illustrates the proportion of genome coverage at various levels in different environments.

### 3.6 Genome divergence between strains of species in each environment

**Figure 8:**
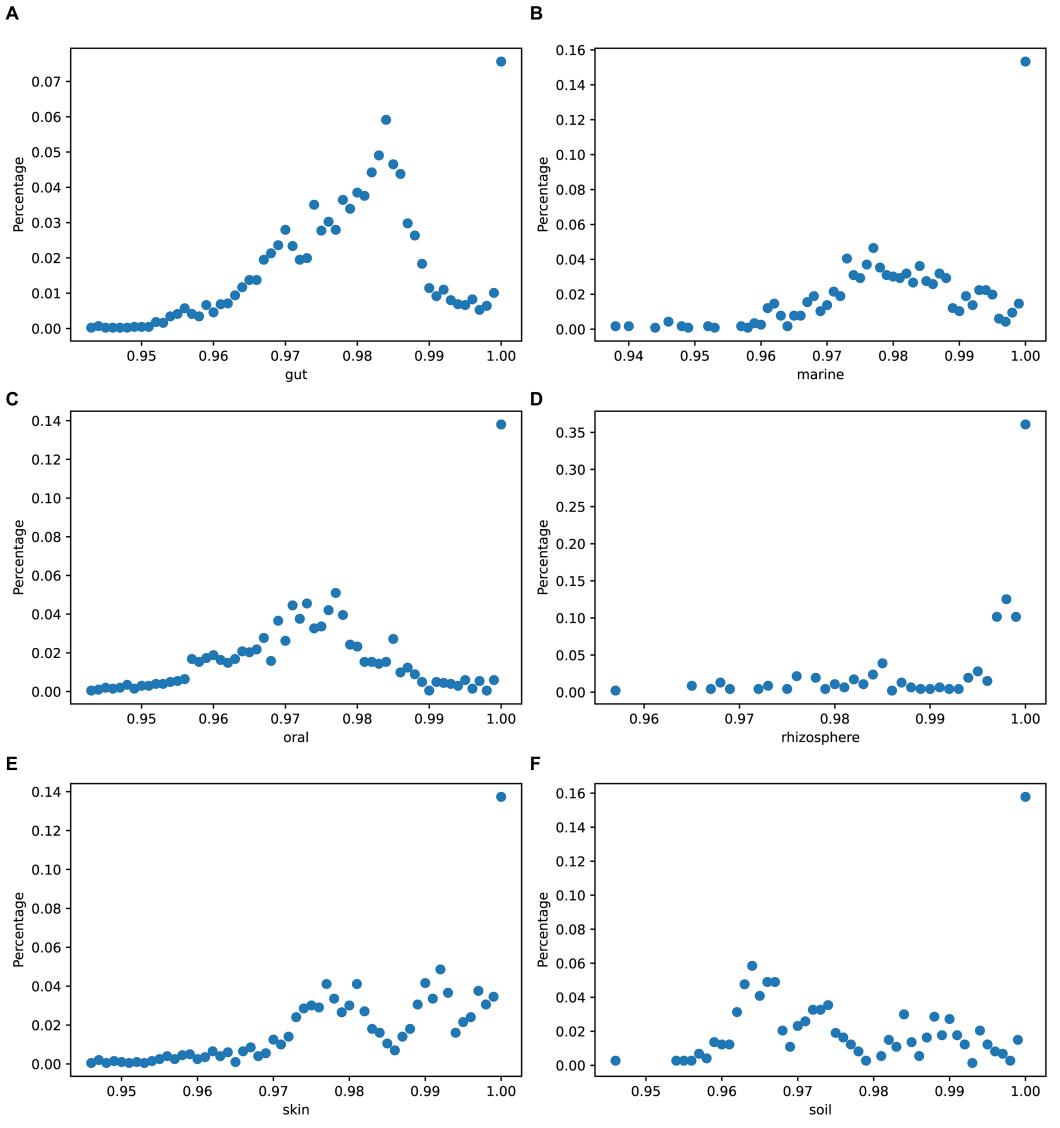
Genome divergence of strains in simulated data. The *x*-axis values represent the numbers obtained by subtracting mash results from 1: **(A)** gut, **(B)** marine, **(C)** oral, **(D)** rhizosphere, **(E)** skin, and **(F)** soil.

To quantify the genetic variation between species, we used mash (Ondov et al., 2016) to obtain the genome divergence of strains in simulated data across different environments. Figure 8 displays variations in the genomic differences among strains of species across different environments.

## 4 Discussion and conclusions

MCSS is a convenient and versatile metagenomic community simulation software that can generate diverse simulated data while ensuring community similarity. MCSS can generate a simulated microbiome based on environmental parameters, learn from user-input sequencing data features, and allow users to specify the microbiome composition. Furthermore, it can simulate the species composition, species abundance, and intra-species heterogeneity of microbiomes, making the simulated communities closely resemble real metagenomic communities. In addition, the generated third-generation sequencing data increases its utility. The mentioned features allow it to cater to various cases of datasets to meet the evaluation needs of metagenomic assembly and analysis tools to help relevant researchers improve their software or algorithms.

## Data availability statement

The original contributions presented in the study are included in the article/supplementary material, further inquiries can be directed to the corresponding authors.

## Author contributions

XH: Data curation, Methodology, Software, Writing – original draft, Writing – review & editing. JY: Methodology, Writing – original draft, Writing – review & editing. JS: Data curation, Writing – review & editing. FL: Methodology, Supervision, Writing – review & editing. WP: Funding acquisition, Methodology, Project administration, Supervision, Writing – review & editing.
